# Two Cases of Placenta Previa

**Published:** 1898-01

**Authors:** Grosvenor R. Trowbridge

**Affiliations:** Buffalo, N. Y.


### Clinical Report.

### TWO CASES OF PLACENTA PREVIA.

BY GROSVENOR R. TROWBRIDGE, A. M., M. D., Buffalo, N. Y.

NOT that I have anything especially new in the treatment of placenta previa to offer do I report the following cases, but more because of the favorable results obtained under unfavorable conditions and surroundings by an expectant and simple treatment. So much has been said and reported by the various authorities in the management of these cases that it would seem impertinent in me to offer any new suggestions. The one fact, however, that I wish to be borne in mind is that both of these cases occurred in a section of our city which is not notorious for its cleanliness and sanitary condition ; and such being the case, I consider myself very fortunate in obtaining the results I did.

CASE I.—Mrs. R., aged 38, had given birth to eight healthy children ; no previous complications in any of her labors. I was called to see her about two weeks before she expected to be confined. There was a profuse hemorrhage and the woman was quite weak from loss of blood. From the history I learned that she had been flowing more or less for about one month and that it had increased to an alarming extent on the day I was called. An examination revealed the fact of a partial placenta previa, with a soft and patulous os dilated to about the size of a five-cent piece. I immediately placed her in bed (she had been up a greater part of the time), with the buttocks well elevated, cleansed the vagina and inserted an antiseptic tampon of gauze, at the same time ordering the triple bromides and morphine in full doses, together with stimulants. I was anticipating that labor would begin almost immediately, but was surprised the same evening to find that the hemorrhage had almost ceased and the woman was in a greatly improved physical condition with no signs or symptoms of labor coming on. I persisted in this treatment, keeping the bowels open with small enemata, and was so far successful that labor did not occur until four days before the expected time. When labor began I stripped off the part of the placenta immediately around the os and, dilating as rapidly as possible, allowed the pressure of the advancing head to act as a tampon. The labor was uneventful, although slow, but with little hemorrhage, and she was delivered of a strong, healthy child. Immediately after the delivery of the child I expelled the placenta by the Credè method, finding some difficulty, as it was quite adherent. Very little hemorrhage occurred after delivery of the placenta, and this, with rest and the use of ergot for a week, was easily controlled. The woman made an uneventful recovery.

CASE II.—Mrs. K., aged 32 years, had given birth to several children without any complications. I was called by the midwife in attendance and found a most profuse hemorrhage, which was due to a central implantation of the placenta. The woman was quite weak, and there was but very little dilation of the os. Labor was due in one week and this had been the first hemorrhage. I tamponed and ordered stimulants and small doses of morphine. This was in the morning. In the afternoon I found that strong labor pains had begun, but no apparent dilation of the os. I immediately tore a hole through the portion of placenta covering the os and stripped it away from the margins. To my surprise the placenta was almost immediately delivered and closely following came the child (breech presentation) very much cyanosed, but alive, and after a half-hour's work was completely resuscitated. There was a profuse hemorrhage for a few moments, but it was controlled by the use of ergotin and manipulation. The woman made an uneventful recovery.

I simply report these two cases, not to advance anything new in their treatment, but to show why I am of the opinion that to allow nature to do all she can without any interference in the way of induced labor and the like will accomplish good results. Considering the fact that the mortality is great in cases of placenta previa, being, I believe, about one to four in the mother's case and one to three in that of the child, I consider myself very fortunate in these two cases.

In cases of placenta previa when the hemorrhage is not great before full term, absolute rest, with the tampon (which should not remain in situ for more than ten hours), will often suffice to prevent labor from occurring before full term. Morphine or opium may be used to quiet labor pains and cool drinks allowed. The bowels must be cared for, best, I think, with enemata. "In case the hemorrhage is severe, labor should immediately be induced and the child and placenta delivered as soon as possible" (Barnes), but the severity of the hemorrhage and the mother's condition, I believe, should be very carefully considered before any radical interference is undertaken.

1331 MAIN STREET.

---

MENTHOL FOR INSECT BITES.—A 10 per cent. solution of menthol in ether is recommended to promptly allay the pain of all kinds of insect stings. A menthol pencil may be used instead.—*Denver Med. Times.*

